# Effect of economic uncertainty on public health expenditure in Economic Community of West African States: Implications for sustainable healthcare financing

**DOI:** 10.1002/hsr2.678

**Published:** 2022-06-16

**Authors:** Chukwunonso Gerald Iheoma

**Affiliations:** ^1^ Statistics Department, Central Bank of Nigeria Abuja Nigeria

**Keywords:** economic growth, economic uncertainty, health expenditure, low income, lower middle income, population growth

## Abstract

This study investigates the dynamic effect of economic uncertainty on public health expenditure in the Economic Community of West African States region. The investigation is motivated by the recent volatilities in the global economy in the face of increasing demand for adequate funding of health systems in the region, and the need to fill the existing gap in the literature. The study employs the panel autoregressive distributed lag model to express the theoretical relationship between public health expenditure per capita, economic uncertainty and population growth rate, and estimates the model parameters using the mean group and the pooled mean group estimators, after accounting for stationarity and cointegration. Results reveal that on the aggregate, economic uncertainty and population growth are significant determinants of per capita health spending in the long run. When the countries are disaggregated by income groups, evidence suggests that in low‐income countries, economic uncertainty is negatively associated with health spending in the short run, while a growing population reduces health spending per capita in the long run. In lower‐middle‐income countries, economic uncertainty increases health spending in the short run, but reduces it in the long run as uncertainty persists, while population growth negatively impacts health spending in the long run. We conclude that the dependence on public funding of the health system in the region appears unsustainable. Thus, health financing policies need to explore alternative funding mechanisms that entrench cost‐sharing between the public and private financiers.

## 1 INTRODUCTION

In developing countries characterized by low levels of investment, income, employment opportunities, life expectancy, and human development index on the one hand, and rising poverty levels on the other hand, public expenditure has become a major policy tool for facilitating the development of a country's human capital (World Health Organization [WHO]^1^), and for spurring the economy unto the path of growth and sustainable development. The thinking among development practitioners is that increased public spending on healthcare should be a priority for governments at all levels. This thinking is motivated by the benefits of adequate funding of the health system for the poor population on the one hand, and the negative macro and microeconomic consequences of inadequate funding of the system on the other hand.

An optimally funded health system ensures improved health status and human capital; reduced burden of out‐of‐pocket (OOP) health expenditure for poor households; and facilitates the development of the human capital required for sustainable development.^2^ On the other hand, suboptimal public health spending can cause severe socioeconomic consequences for poor households due to high cost of accessing health services from private providers. High cost of healthcare could result in catastrophic OOP health expenditure as households are forced to pay a large share of their income on health services, pushing some into poverty, and others into even deeper poverty than they are already in. Households seeking health services may be forced to borrow money, sometimes at very high‐interest rates, or to sell their assets, to pay for health services. The alternative for such households is to forgo health services and live with their illness and suffer the short‐ and long‐term consequences.^3^

A major challenge of public health financing in developing countries is the impact of global economic uncertainty on domestic health budget. Economic uncertainty is one of the inevitable features of a globalizing world. The macro and microeconomic impacts of economic uncertainty have been a source of concern to policymakers especially in developing countries that mostly suffer the severe impacts of the phenomenon. There are concerns that reduction in public health spending due to economic uncertainty could affect the poor and vulnerable populations and, in turn, erase the development progress that has been made thus far. Addressing existing poor health outcomes within the context of economic crisis has become a policy priority given the importance of a healthy population in a developing country.^4^ Also, understanding how a growing population affects health expenditure is critical for evolving sustainable health financing policies. This is because evidence suggests that a growing population can increase the fiscal burden of health expenditure on the government.^5^, ^6^, ^7^

Various studies have attempted to understand how economic uncertainty impact public spending on healthcare. An examination of the impact of economic crisis on healthcare resources in the Eastern Mediterranean countries of WHO reveals that being unemployed and having to spend from OOP are negatively correlated with healthcare expenditure per capita: a 1% rise in unemployment is found to decrease health expenditure per capita by $138, and an increase of 1% in OOP is associated with a $12 decrease in per capital health expenditure.^8^ In another study that examined the health impact of the 2007–2009 economic recession, evidence suggests that macro and human unemployment impacts of the recession were associated with declining fertility, increasing morbidity, psychological distress, and suicide.^9^, ^10^ In Nigeria, Rufai et al.,^11^ finds that when economic recession leads to an increase in the prices of inputs for household enterprises, and loss of job, household health expenditure will be adversely affected.

By reducing or eliminating income, economic uncertainty negatively impacts the willingness and ability to purchase healthcare using personal income. A study of the impact of economic recession on residents' willingness to make OOP payment for healthcare in 60 countries between 2000 and 2016 finds that economic recession inhibited the willingness to make OOP, even after controlling for country‐specific differences. This happens because uncertainty reduces employee compensation in most countries.^12^ A study of the impact of the Greek financial crisis finds that the population level trend in household health spending was reversed after the crisis began. OOP health spending, and the poorest households received a disproportionately larger impact of the crisis.^13^

Increased public spending on healthcare has been found to cushion the adverse effect of economic uncertainty on health outcomes. A study of 5556 municipalities in Brazil finds that although the recessions increased mortality rate, increased health and social protection expenditure seemed to mitigate detrimental health effects of the recessions, especially among vulnerable populations. In municipalities with high public expenditure on health and social protection programmes, no significant increases in recession‐related mortalities are observed.^14^ In European countries, evidence suggests that the prevalence of strong social safety nets appears to have moderated the negative health impact of the 2007–2009 economic recession.^9^

The reaction of health expenditure to economic uncertainty tends to be determined by the level of development of a country. In developed countries, health expenditure is found to be counter‐cyclical, while it is procyclical in developing economies.^15^ An investigation of the effect of business cycle on health expenditure in China between 2002 and 2018 shows that after the 2008 financial crisis, influence of business cycles on health expenditure shifted from being procyclical to being counter‐cyclical, indicating increased budgetary allocation to healthcare.^16^

The objective of this study is to determine the effect of economic uncertainty on public per capita health spending in the Economic Community of West African States (ECOWAS). This determination is important from various perspectives. First, public spending is a major healthcare services input in the ECOWAS region. As a commodity exporting region, the revenue of member States, and the public health budget that depends on it, could be negatively affected by uncertainty in the global economy. Second, the growing levels of poverty in the region, leading to decreased household health expenditure, and the poor state of health insurance imply that the poor and vulnerable populations will have to rely on government health facilities to access affordable healthcare, the funding of which may be vulnerable to economic uncertainty, and third, achieving the third goal of the sustainable development goals (SDGs)—good health and wellbeing—is dependent on improved funding of the health system, indicating the need to understand how public health financing is affected by economic uncertainty. Equally, achieving this objective will provide empirical answers to the question: what is the effect of economic uncertainty on public health spending in the ECOWAS region? These provide compelling justifications for policymakers to understand how public spending on healthcare is impacted by economic uncertainty. The study contributes to existing literature on the sustainability of public health financing amid uncertain regional economic outlook by highlighting how public health financing could be impacted by economic uncertainty, as well as the magnitude of such impact.

ECOWAS is a regional economic group comprising of all the 15 countries that make up the West African region, with a mandate of promoting economic integration. The Commission was set up to foster the ideal of collective selfsufficiency for its member states and works to harmonize macroeconomic policies toward achieving regional economic integration.^17^ In 2001, African Union heads of state, including those of ECOWAS countries, pledged to allocate at least 15% of their annual government budget to the health sector under the Abuja Declaration. This commitment marks an important initiative in the history of public health financing in the region.^18^

However, evidence reveals that between 2010 and 2018, no West African country attained the 15% threshold for public budgetary allocation to the health sector. The top three countries in this regard, Ghana (8.43%), Carbo Verde (8.29%), and Burkina Faso (7.60%), still allocates less than 10% of their total annual budget. Other countries, for example, Guinea (3.05%), Liberia (3.46%), and Guinea‐Bissau (3.56%) spend less than 5% of their annual budget.^19^ Given this scenario and coupled with the COVID‐19‐induced global economic uncertainty that has affected the economies of the countries of the region, this study becomes timely and policy‐relevant.

As far as we know, no study on this issue has been undertaken in the region. The closest approximation to this study is the work by Aregbeyen and Akpan.^10^ However, their study differs from this study in scope and methodology. Their study adopts a micro‐outlook and considers the effect of economic shock on health expenditure of rural households using the Heckman's selectivity model. Our study focuses on the macro dynamics of economic uncertainty on public health spending. This is important because of the growing demand on the government for increased health budget for a poor, growing population. Methodologically, we adopt the panel autoregressive distributed lag (PARDL) model. This model allows us to determine the dynamic effect of economic uncertainty on public health expenditure in the short and long run, while accounting for group‐specific effect.

The rest of the study is structured as follows: immediately following this introduction is Section 2, containing the details of the data and method. In Section 3, we present the estimated results and their interpretations. Section 4 contains the conclusion and policy implications of the study.

## 2 MATERIALS AND METHODS

### 2.1 Data sources

This is a macroeconomic panel data study that uses data from the 15 countries in the ECOWAS region for a period of 19 years (2000–2018), totaling 285 observations. Period of coverage is determined by data availability. The variables used for the study are economic uncertainty (computed from growth rate of gross domestic product), public health expenditure per capita, and population growth rate. Data on public health expenditure are obtained from the WHO's online database. Data on economic, and population growth rates are extracted from the World Bank's world development indicators (WDI) online database.

### 2.2 The model

Various theories provide broad framework for understanding the linkage between growth in government expenditure caused by increasing demand for social services. The seminal work of Adolph Wagner—Wagner's hypothesis of increasing public debt—explains that government expenditure is bound to grow in an industrializing economy as the government strives to provide basic infrastructure required to support industrialization. One of the major arguments of the law is that because the goods and services (e.g., healthcare) supplied by the public sector have high‐income elasticity of demand, as this elasticity increases, public expenditure must increase proportionally to the increase in income.^20^ Musgrave hypothesis attributes the growth in government expenditure to structural adjustments that coincide with the development process of a country. In the initial phase of development, private capital formation is low, while population continues to grow. This leads to reliance on publicly provided goods and services, leading to the burgeoning of public budget.^21^

The Brown and Jackson^22^ microeconomic theory of public expenditure considers the demand side factors (taste and income) and the supply side factors (tax rate, and cost of production) to determine factors influencing public expenditure growth. The model recognizes the service environment, population growth—leading to increased demand for healthcare services—and changes in the quality of public goods demanded by the median voter as determinants of growth in public expenditure. This study adopts the Brown and Jackson microeconomic theory of public expenditure as the framework for its model, and hypothesis that public health expenditure in the ECOWAS is affected by economic uncertainty. Various studies have employed the Brown and Jackson framework. They find population and economic growth as determinants of public expenditure.^10^, ^23^, ^24^ The innovation of this study is the introduction of economic uncertainty into the model as one of the determinants of public health spending. The PARDL model for consideration is given as:

(1) $$△{Χ}_{it}=\alpha_{i}+\sum_{l=1}^{k}\gamma_{i}{Χ}_{i,t-1}+\sum_{l=1}^{k}\lambda_{i}{ϒ}_{i,t-1}+\sum_{l=1}^{k}\phi_{i}{Ζ}_{i,t-1}+\varepsilon_{it},$$

*l* = 1, 2, 3, …, *k*; *t* = 1, 2, 3, …, *T*.

In Equation (1), ${Χ}_{it}$, ${ϒ}_{it}$, ${Ζ}_{it}$ are health expenditure per capita, economic uncertainty, and population growth rate, respectively for individual countries. $\gamma_{i}$, $\lambda_{i}$, $\phi_{i}$ are the corresponding long‐run parameters of the variables, $\varepsilon_{it}$ is the disturbance term, *i* and *t* represent country and time respectively, while *k* indicates the optimal lag length. The model is justified as the period of coverage (*T* = 19) is larger than the number of cross sections (*N* = 15).^25^

The model assumes that current health expenditure is, to a reasonable extent, dependent on its previous value. Government officials do compare the previous period's expenditure with the current period's target to identify funding gaps. Current expenditure is determined based on this information. There exists a link between economic uncertainty and health expenditure in the literature. During periods of economic shocks, government's reaction to its expenditure on health could be positive or negative depending on its health and human development policies. If economic uncertainty leads to reduction in government revenue, for example, health spending is likely to fall if healthcare is not a priority policy area. On the other hand, if healthcare is a major policy thrust of the government, health spending may remain stable as the government reallocates funding from low priority areas to the health system.

Population growth is expected to be positively correlated with health spending. A growing population can be associated with increased demand for healthcare. Public spending on health must increase proportionately to meet this demand. Evidence suggests that a growing population can increase the fiscal burden of health expenditure on the government. For example, the study by Korwatanasakul et al.^5^ finds that demographic transition increases public health expenditure. Other studies confirm that increase in ageing population induces a relatively strong reaction from health expenditure per capita.^6^, ^7^

The model estimation begins with panel unit root tests on the variables. We use the Im‐Pesaran‐Shin (IPS) test (Im et al. 2003),^26^ and the Levin‐Lin‐Chu (LLC) (Levin et al)^27^ unit root test techniques. Both tests assume cross‐sectional independence, and that all series are nonstationary under the null hypothesis.^28^, ^29^ The selection of the lag length is by the Bayesian‐Schwarz criterion. Next, we test for the existence of long‐run relationship between the variables using the Pedroni and Westerlund cointegration tests. Both tests assume panel‐specific cointegrating vectors where all panels have individual slope coefficients. The test statistics are derived from a model in which the autoregressive (AR) parameter is either panel‐specific or is the same over the panels. Panel‐specific‐AR tests the null hypothesis of no cointegration in some panels, while the AR that is same across the panels tests the null hypothesis of no cointegration for all panels.^30^, ^31^, ^32^

Testing for cointegration is a necessary step to establishing if variables empirically exhibit meaningful long‐run relationships.^33^ If we do not reject the null hypothesis, we conclude that the variables are devoid of any long‐run relationships, in which case we restrict our analysis and interpretation to the short‐run estimation. Conversely, if we do not accept the null hypothesis, we reparameterize Equation (1) into an error correction model. Doing so produces a model that incorporates the short and the long‐run information regarding the interaction of the variables, as well as the error correction mechanism.

(2) $$\begin{array}{l} △{Χ}_{it}=\alpha_{i}+\rho_{i}{Χ}_{i,t-l}+\sum_{l=1}^{k}\delta_{1}△{Χ}_{i,t-l}+\sum_{l=1}^{k}\delta_{2}△{ϒ}_{i,t-l}+\sum_{l=1}^{k}\delta_{3}△{Ζ}_{i,t-l}+\gamma_{i}{Χ}_{i,t-l}+\lambda_{i}{ϒ}_{i,t-l}+\phi_{i}{Ζ}_{i,t-l}+\varepsilon_{it}, \end{array}$$

*l* = 1, 2, 3, …, *K*; *t* = 1, 2, 3, …, *T.*

Where all other variables and parameters remain as previously defined. $\rho_{i}$ captures the speed of adjustment to long‐run equilibrium. It is derived as the error term from Equation (1) whose coefficients are obtained by normalizing the equation on Δ${Χ}_{it}$(Nkoro & Uko, 2016). Δ is the first difference operator, indicating that the variables were first differenced for short‐run analysis, while $\delta_{1}$, $\delta_{2}$, and $\delta_{3}$ are the short‐run parameters of the model. We expect the speed of adjustment to be negatively signed to indicate long‐run convergence of the model.

Economic uncertainty is estimated using a stochastic model: the generalized AR conditional heteroscedasticity (GARCH) model.^34^ From the general form of GARCH (p,q) model, the GARCH (1,1) model can defined as:

(3) $${ϒ}_{it}=C+\psi {ϒ}_{it-1}+\pi \sigma_{it-1},$$

(4) $$\sigma_{it}^{2}=\eta +\beta \sigma_{it-1}^{2}+\alpha \varepsilon_{it-1}^{2}.$$

Equation (3) is the mean equation. The dependent variable (${ϒ}_{it}$) is economic growth measured by the growth rate of the gross domestic product. *C* is the constant, while $\sigma_{it-1}$ is the error term. After estimating Equation (3), we extract the residuals and use them as the dependent variable in Equation (4). Equation (4) assumes that the squared residual from the preceding equation is a function of its lagged value and a random innovation ($\varepsilon_{t-1}^{2}$). After estimating Equation (4), we extract the series of the random innovation and use that as our ${ϒ}_{it}$ in Equations (1) and (2).

We use the mean group (MG) and the pooled mean group (PMG) estimators to determine the relationship between the variables. The PMG restricts the long‐run estimates to be equal across countries, while allowing them to differ in the short run. The short‐run relationship captures country‐specific heterogeneity which may arise from the unequal magnitude of economic shock for each country. Conversely, The MG estimator allows for heterogeneity in the short and long run relationships.^28^

The Hausman test is used to select the optimal estimator. By default, the Hausman test considers the PMG as the null hypothesis and the MG as the alternative. The statistical significance of the test implies the rejection of the PMG estimates, allowing for the acceptance and interpretation of the MG estimates. Where the test is insignificant, the PMG estimates are favored.^35^

In the postestimation, we test for the presence of cross‐sectional dependence of the error terms of the estimated equations using the Pesaran et al.,^36^ cross‐section dependence (CD) test. This test is important because ignoring the presence of cross‐sectionally dependence in the error terms can decrease estimation efficiency to the extent that the panel estimator may provide little gain over the single‐equation ordinary least squares.^37^ Pesaran^38^ shows that the test is consistent even with small *N* and *T*, and compares favorably with the Breush‐Pagan Langrage Multiplier test. Estimations are done using STATA 15 software. The estimated coefficients are considered statistically significant if the *p*‐value is less than 0.05.

To determine whether the effect of economic uncertainty is dependent on national income levels, we disaggregate the countries by income groups according to the World Bank country classification. Countries with income below $1045 are classified as low‐income countries (LICs), those with income between $1046 and $4095 are lower‐middle‐income countries (LMICs). Countries whose income exceeds $4,096 but is below $12,695 belong to the upper‐middle‐income group, while a country is regarded as high income if the income level exceeds $12,696.^39^, ^40^ Of the 16 countries in the ECOWAS region, nine are classified as LICs^1^, while six belong to the LMICs^2^ group.

### 2.3 Empirical results

In Table 1, we present the summary statistics of our variables, together with the confidence intervals (CI) of the means. Per capita health expenditure averages $13.99 for all countries. However, there is substantial disparity in spending among individual countries: while some countries spend as low as $1.13, others spend over 900% of regional average ($117.20). Public health spending is particularly lower in LICs than in LMICs: the average per capita spending in the former is $6.08, while it is $25.89 in the latter, indicating huge divergence. The maximum spending in LICs ($19.23) translates to just about 16.4% of maximum spending in LMICs ($.117.20), although spending is more volatile in LMICs (SD: 30.24) than in LICs (SD: 3.45).

**Table 1:** Summary statistics of study variables

Country classification/variable	Mean	Confidence interval of mean	SD	Min.	Max.	Obs.
**Panel A: All countries**						
Health exp. per capita	13.99	11.48−16.51	21.57	1.13	117.20	285
Economic uncertainty	4.71E‐08	−11.47 to 11.47	93.02	−36.07	1175.44	255
Population growth rate	2.69	2.62−2.76	0.62	1.16	5.36	285
**Panel B: LICs**						
Health exp. per capita	6.08	5.56−6.60	3.45	1.13	19.23	171
Economic uncertainty	6.48	−12.49 to 25.4	118.79	−36.07	1175.44	153
Population growth rate	2.90	2.81−2.99	0.59	1.7152	5.36	171
**Panel: LMICs**						
Health exp. per capita	25.86	20.25−31.47	30.24	3.25	117.2	114
Economic uncertainty	−9.72	−13.49 to 5.95	19.20	−21.47	92.57	102
Population growth rate	2.38	2.28−2.47	0.51	1.16	3.04	114

Abbreviations: LIC, low‐income countrie; LMICs, lower‐middle‐income countries

Our indicator of economic uncertainty differs markedly between both income groups. It averages 6.48% in LICs, indicating predominantly positive changes in the growth rate of GDP in those countries. Conversely, LMICs seem to have recorded largely negative movements in their rates of GDP growth (−9.72%) over the period covered by this study. An average population growth rate of 2.69% is observed in the region. Maximum population growth rates of 5.36% and 3.04% were recorded in LICs and LMICs respectively, and the rates are relatively stable in both groups.

Table 2 shows the results of the unit root tests conducted on the variables. The IPS and LLC tests results tend to follow a similar pattern on the aggregate. For all countries, at level, both tests indicate that economic uncertainty and population growth are stationary, while health expenditure per capita is nonstationary. However, at first difference, all variables appear stationary in both tests. Similar results are observed when the countries are disaggregated by their income groups.

**Table 2:** Panel unit root test of the variables

Variable	IPS		LLC	
Panel A: All countries	Level	First diff	Level	First diff
Health expenditure per capita	1.2527	−10.7101***	−1.1163	−11.9845***
Economic uncertainty	−14.1057***	−21.7939***	−18.9870***	−25.8584***
Population growth rate	−8.9137***	−10.8497***	−8.8720***	−11.5835***
Observations	285	285	285	285
**Panel B: LIC**				
Health expenditure per capita	1.6417	−7.9463***	−0.6712	−8.6940***
Economic uncertainty	−8.1678***	−17.0078***	−10.2836***	−20.6395***
Population growth rate	−6.7855***	−11.3403***	−6.6209***	−12.0576***
Observations	171	171	171	171
**Panel C: LMIC**				
Health expenditure per capita	−0.0301	−7.2105***	−0.8788	−8.3169***
Economic uncertainty	−12.2997***	−13.6314***	−15.8397***	−16.0833***
Population growth rate	−5.7833***	−3.2427***	−6.5946***	−3.7912***
Observations	114	114	114	114

*Note*: ***, **, * denote the rejection of the null of a unit root for 99%, 95%, and 90% significant levels respectively.

Abbreviation: IPS, Im‐Pesaran‐Shin; LLC, Levin‐Lin‐Chu; LMICs, lower‐middle‐income countries.

The cointegration test results in Table 3 indicate that Pedroni and Westerlund tests are all significant at various levels for all countries. Both tests are equally significant for LICs, while only the Pedroni test is significant for the LMICs. Thus, we fail to accept the null hypothesis of no cointegration and conclude that the variables share long‐run relationship. Additionally, the error correction parameters in the various regression results are negatively signed and less than unity, further confirming the existence of long‐run relationship among the variables.

**Table 3:** Panel cointegration tests

	All countries		LICs		LMICs	
Panel A: Pedroni test	Statistic	*p*‐Value	Statistic	*p*‐Value	Statistic	*p*‐Value
Modified Phillips‐Perron *t*	2.499***	0.006	2.307***	0.010	1.430*	0.076
Phillips‐Perron *t*	−1.443*	0.074	−1.098	0.136	−0.956	0.169
Augmented Dickey‐Fuller *t*	−1.674**	0.047	−1.079	0.140	−1.325*	0.093
**Panel B: Westerlund test**						
Variance ratio	1.896**	0.029	2.8834***	0.002	−0.5335	0.2968

*Note*: ***, **, * denote the rejection of the null hypothesis of no cointegration for 99%, 95%, and 90% significant levels respectively.

Abbreviations: LIC, low‐income country; LMICs, lower‐middle‐income countries.

Our regression analysis reveals mixed results for the entire panel, as shown in Table 4. The results from the MG estimations are not significant both in the short and long run. Conversely, PMG estimates show that economic uncertainty and population growth are significant determinants of health expenditure only in the long run, although the Hausman test favors the MG. Economic uncertainty reduces health expenditure per capita by 0.12% for a 1% increase, while population growth increases it by 11.50% for a change of similar magnitude. The error correction coefficients in both estimators are properly signed and significant, collaborating the results of the cointegration tests, and confirming the existence of a long‐run relationship among the variables. Speed of adjustment is higher in MG than in PMG. About 51.5% of the discrepancy between the short and the long‐run equilibrium is eliminated per year in the MG estimation, while slightly above a quarter (28.5%) of the disequilibrium is adjusted per year in the PMG.

**Table 4:** Regressions results for all countries

Variable	MG			PMG	
**Long run**					
Economic uncertainty	−0.0610	(0.1280)		−0.1216***	(0.0010)
Population growth rate	−8.8306	(0.2650)		11.4980***	(0.0000)
**Short run**					
Economic uncertainty	0.0216	(0.1560)		0.0169	(0.2320)
Population growth rate	−5.1165	(0.7510)		0.0093	(0.9990)
Error correction	−0.5147***	(0.0000)		−0.2849***	(0.0010)
Constant	17.6828*	(0.0590)		−3.8664	(0.1490)
CD	−0.1330	(0.8940)		0.2620	(0.7930)
Hausman test				3678.74***	(0.0000)
Observations	240			240	

*Note*: ***, **, * denote the rejection of the null hypothesis of no effect at 99%, 95%, and 90% significant levels respectively. Figures in parenthesis are the *p*‐values.

Abbreviations: MG, mean group; PMG, pooled mean group.

Results from the LICs (Table 5) reveal the significant role of population growth in influencing public health expenditure per capita in the long run. Population growth rate is negatively and significantly associated with per capita health expenditure in both the MG and PMG estimators. In the MG, health spending per capita is reduced by 10.41% for every 1% increase in population growth rate. This further reduces by 14.83% in the PMG. In the short run, economic uncertainty and population growth rate are rightly signed but insignificant in the MG, while economic uncertainty retains the expected sign and is significant in the PMG, impacting per capita health expenditure by −0.022% for a 1% change in the uncertainty index. The error correction mechanism appears as expected in terms of magnitude and sign in both estimators, indicating that 41.2% and 26.2% (for MG and PMG, respectively) of the short‐ and long‐run disequilibrium is corrected per year. The Hausman test confirms preference for the PMG estimator.

**Table 5:** Regressions results for LICs

Variable	MG		PMG	
**Long run**				
Economic uncertainty	−0.0199	(0.6730)	0.0116	(0.2590)
Population growth rate	−10.4085*	(0.0840)	−14.8334***	(0.0000)
**Short run**				
Economic uncertainty	−0.0116	(0.1740)	−0.0223***	(0.0290)
Population growth rate	−9.6026	(0.3380)	−7.0462	(0.5290)
Error correction	−0.4120***	(0.0000)	−0.2623***	(0.0030)
Constant	18.9349***	(0.0090)	14.0674***	(0.0020)
CD	−0.2160	(0.8290)	−0.330	(0.7420)
Hausman test			1.0000	(0.6079)
Observations	144		144	

*Note*: ***, **, * denote the rejection of the null hypothesis of no effect at 99%, 95% and 90% significant levels respectively. Figures in parenthesis are the *p*‐values.

Abbreviations: LIC, low‐income countrie; MG, mean group; PMG, pooled mean group.

Table 6 presents the results of the regression analysis for LMICs. Economic uncertainty is a significant long‐run determinant of public health expenditure per capita in LMICs in both estimators, while population growth rate is only significant in the PMG. The impact of economic uncertainty on per capita expenditure is negative; reducing it by 0.12% in the MG, and by 0.26% in the PMG for a unit increase in uncertainty. A 7.96% negative change is observed in per capita health expenditure for a unit change in population growth rate.

**Table 6:** Regression Results for LMICs

Variable	MG		PMG	
**Long run**				
Economic uncertainty	−0.1234*	(0.0730)	−0.2575***	(0.0000)
Population growth rate	−6.4637	(0.7300)	−7.9608**	(0.0450)
**Short run**				
Economic uncertainty	0.0713***	(0.0040)	0.0934***	(0.0010)
Population growth rate	1.6127	(0.9680)	4.0201	(0.8190)
Error correction	−0.6687***	(0.0000)	−0.4497***	(0.0020)
Constant	15.8047	(0.4740)	18.1542***	(0.0000)
CD	−1.0880	(0.2770)	−0.8720	(0.3830)
Hausman test			27.8300***	(0.0000)
Observations	96		96	

*Note*: ***, **, * denote the rejection of the null hypothesis of no effect at 99%, 95%, and 90% significant levels respectively. Figures in parenthesis are the *p*‐values.

Abbreviations: LMICs, lower‐middle‐income countries; MG, mean group; PMG, pooled mean group.

In the short run, only economic uncertainty significantly impacts health spending, increasing it by 0.071% and 0.093% in the MG and PMG respectively for a 1% increase in the uncertainty index. The speed of adjustment in disequilibrium is higher in the MG (66.9%) than in the PMG (45.0%), and the Hausman test result indicates that the MG estimations are more consistent.

## 3 DISCUSSION

The negative effect of economic uncertainty on health spending per capita in LICs manifests in the short run, indicating their vulnerability to economic shocks, and their weak capacity to absorb shocks. Thus, the impact of economic uncertainty is quickly observed in government revenue. Economic uncertainty reduces the size of government revenue from taxes and foreign trade as investment, production, consumption, and trade are slowed down during periods of uncertainty, while unemployment rises. Reduction in government revenue could imply reduced budgetary allocation to the health sector, the result of which is a decline in the per capital health expenditure. The narrative is applicable to the LMICs. The only difference being that due to their higher‐income relative to the LICs, their economies possess the capacity to absorb the negative impact of economic uncertainty at least in the short run. As uncertainty persists in the long run, this capacity is significantly diminished, allowing uncertainty to negatively impact public per capita health spending.

The findings on the effect of economic uncertainty on public per capita health expenditure corroborate that of Faramarzia et al.,^8^ in Eastern Mediterranean countries, and^10^ in Nigeria. They find that economic uncertainty resulting to rise in unemployment and consumer prices reduces per capita health expenditure by reducing the size of revenue available to the government to allocate to the health system.

A growing population is expected to impose huge financial burden on the government as the demand for healthcare increases. This increased demand is expected to lead to increased government funding of the health system as the government tries to cater for its teeming population. However, this is not the case in LICs and LMICs in the ECOWAS region. The effect of population growth on health expenditure per capita in these countries in the long run reflects their low national income level that results in constant or declining health budget in the face of a growing population. The growing population implies that individuals will be struggling for the existing, or possibly depleting public resources. This reduces the amount of health budget available per individual. The implications of reduced health spending per capita include low health status leading to low productivity, high OOP healthcare expenditure leading to low household savings and investment, high mortality rate, and reduced public revenue from taxes as productivity and investment are abysmally low or nonexistent. All these implications interact to reinforce the low‐income status of these countries.

Our results on the effect of population growth on public health expenditure per capita differ from those of similar studies in other contexts. In Europe, Christiansen et al.^7^ find that a growing population can increase the fiscal burden of health expenditure on the government. Other studies confirm that increase in aging population is positively associated with per capita health expenditure in China^6^ and in Southeast Asia.^5^ The reason for this difference in results is rather intuitive: a growing population leads to increased demand for public health services, especially in poor economies where household income is inadequate to satisfy the household healthcare needs. Where health expenditure does not grow proportionally to population growth, the size of the public health budget available per individual declines.

The study is limited to the effect of economic uncertainty on public health expenditure due to the availability of comprehensive data set on that aspect of healthcare financing. Further studies may consider other forms of healthcare financing such as private health expenditure and external health expenditure. Also, investigation of the effect of economic uncertainty on household health spending may be an innovative research area. Other factors affecting health expenditure other than economic uncertainty may also be explored in subsequent research.

## 4 CONCLUSION

This study aims to investigate how uncertainty in the global and national economies affect the health budget in ECOWAS countries. This is motivated by the recent global economic shocks arising from the discovery of Corona Virus disease on the one hand, and the increasing public dependence on the government for provision of quality, affordable, and accessible healthcare, on the other hand. The study stands out from similar studies as it focuses on the macro dynamics of economic uncertainty on public health spending. This is important because of the growing demand on the government for increased health budget for a poor, growing population. The STUDY contributes to existing literature on the sustainability of public health financing amid uncertain regional economic outlook. The evidence from the study is expected to guide policymakers in formulating sustainable health system financing policies.

The study analyses data on health expenditure per capita, economic uncertainty indicator, and population growth rates from the 16 countries that make up the region using the PARDL approach. This approach addresses the research questions by regressing economic uncertainty on public health expenditure, while accounting for short and long‐run effects. The study answers the question: what is the effect of economic uncertainty on public health spending in the ECOWAS region? Before estimating the model, preliminary tests are conducted to check for stationarity and cointegration. Results reveal that the negative effect of economic uncertainty in LICs is transient—occurring only in the short run. In LMICs, its effect is negative in the short run, but positive in the long run. The effect of population growth is more noticeable in the long than in the short run in both income groups.

The results highlight the imperatives of re‐evaluating the existing health system funding models in the countries of the ECOWAS region in view of the inevitable effect of global economic fluctuations on the revenue base of those countries. A sustainable funding model may require exploring the private–public partnership options, introducing, and strengthening health insurance schemes, as well as considering other funding models that incorporate cost‐sharing.

## AUTHOR CONTRIBUTIONS

**Chukwunonso Gerald Iheoma**: Conceptualization; data curation; formal analysis; methodology; writing—original draft; writing—review and editing.

## CONFLICT OF INTEREST

The author declares no conflict of interest.

## TRANSPARENCY STATEMENT

The lead author (manuscript guarantor) affirms that this manuscript is an honest, accurate, and transparent account of the study being reported; that no important aspects of the study have been omitted; and that any discrepancies from the study as planned (and, if relevant, registered) have been explained.

## Data Availability

The data underlying this article are available in WHO global health observatory,^41^ at https://www.who.int/data/gho/data/indicators/indicator-details/GHO/current-health-expenditure-(che)-per-capita-in-us$; and WDI databank at https://databank.worldbank.org/source/world-development-indicators

## ENDNOTES

1 Burkina Faso, Gambia The, Guinea, Guinea Bissau, Liberia, Mali, Niger, Sierra Leone, and Togo.

2 Benin, Cabo Verde, Cote d'iovre, Ghana, Nigeria, and Senegal.
